# Bioinformatics Analysis Identifies Key Genes and Pathways in Acute Myeloid Leukemia Associated with DNMT3A Mutation

**DOI:** 10.1155/2020/9321630

**Published:** 2020-11-23

**Authors:** Shuyi Chen, Yimin Chen, Jielun Lu, Danyun Yuan, Lang He, Huo Tan, Lihua Xu

**Affiliations:** ^1^Department of Hematology, The First Affiliated Hospital of Guangzhou Medical University, Guangzhou, Guangdong 510000, China; ^2^Department of Urology & Minimally Invasive Surgery Center, The First Affiliated Hospital of Guangzhou Medical University, Guangdong Key Laboratory of Urology, Institute of Urology, Guangzhou, Guangdong, China; ^3^Department of Pediatrics, The First Affiliated Hospital of Guangzhou Medical University, Guangzhou, Guangdong 510000, China

## Abstract

**Background:**

DNA methyltransferase 3 alpha (DNMT3A) mutation was one of the most frequent genetic alterations in acute myeloid leukemia (AML), which was associated with poor prognosis and appeared to be a potential biomarker. Herein, we aimed to identify the key genes and pathways involved in adult AML with DNMT3A mutations and to find possible therapeutic targets for improving treatment.

**Methods:**

The RNA sequencing datasets of 170 adult AML patients were obtained from The Cancer Genome Atlas (TCGA) database. EdgeR of the R platform was used to identify the differentially expressed genes (DEGs). Gene Ontology (GO) and Kyoto Encyclopedia of Genes and Genomes (KEGG) enrichment analyses were performed by Metascape and DAVID. And protein-protein interaction (PPI) network and clustering modules were analyzed with the STRING database and Cytoscape software.

**Results:**

Mutated DNMT3A resulted in a shorter overall survival (OS) in AML patients and obviously associated with age, blast percentage in peripheral blood, and FLT3 mutation. A total of 283 DEGs were detected, of which 95 were upregulated and 188 were downregulated. GO term analysis showed that DEGs were significantly enriched in neutrophil degranulation, myeloid cell differentiation, stem cell proliferation, positive regulation of neurological system process, leukocyte migration, and tissue morphogenesis. KEGG pathway enrichment analysis indicated that the pathway of cancer, PI3K-Akt signaling pathway, and transcriptional misregulation in cancer may play a crucial role in DNMT3A mutation AML. Seven hub genes (BMP4, MPO, THBS1, APP, ELANE, HOXA7, and VWF) had a significant prognostic value.

**Conclusion:**

Bioinformatics analysis in the present study provided novel targets for early diagnosis and new strategies for treatment for AML with DNMT3A mutation.

## 1. Introduction

Acute myeloid leukemia (AML) is a common type of hematopoietic neoplasm characterized with molecular characteristics [1]. Molecular analyses of leukemic blasts from AML patients have suggested that there was an obvious heterogeneity in the presence of acquired gene mutations and changes in gene and microRNA expression. Multiple submicroscopic genetic alterations with prognostic and therapeutic implications have been discovered, including mutations in nucleophosmin 1 (NPM1), CCAAT-enhancer-binding protein alpha (CEBPA), Fms-like tyrosine kinase 3 (FLT3), and DNA methyltransferase 3 alpha (DNMT3A) [2, 3]. In particular, NPM1, FLT3, and DNMT3A mutations are the most frequent genetic alterations in AML [3–6].

As an epigenetic modification, DNA methylation is important for mammalian development. Three genes (DNMT1, DNMT3A, and DNMT3B) have been found to encode proteins with DNA methyltransferase activity [7, 8]. However, mutations of DNMT3A have been broadly detected in various cancers [9–12], especially in adult hematologic malignancies [3, 13–15]. Associated with increased adverse outcome, DNMT3A mutation has been identified as a biomarker for prognostic evaluation and minimal residual disease (MRD) monitoring in AML [16–18]. Therefore, the aim of the research was to identify the key genes and pathways in AML with DNMT3A mutation using bioinformatics analysis.

## 2. Materials and Methods

### 2.1. RNA Sequencing Data

Information of 170 adult AML patients, including RNA-seq dataset, corresponding survival profiles, and gene mutation information, was obtained from The Cancer Genome Atlas (TCGA) database (https://gdc-portal.nci.nih.gov/) [1] and downloaded from UCSC Xena (http://xena.ucsc.edu/).

### 2.2. Identification of Differentially Expressed Genes (DEGs)

EdgeR was used to screen DEGs between DNMT3A mutation and wild-type AML patients according to the user's guide [19, 20]. DEGs were identified with the cut-off value of log_2_  | fold change (FC) | ≥1 and *P* value < 0.05. A heat map and volcano plot of DEGs were drawn by the ggplots package in the R platform.

### 2.3. Enrichment Analysis of DEGs

Gene Ontology (GO) term enrichment was analyzed by Metascape (http://metascape.org), including biological process (BP), molecular function (MF), and cellular component (CC) [21], while the Kyoto Encyclopedia of Genes and Genomes (KEGG) pathway enrichment was analyzed by DAVID (https://david.ncifcrf.gov/tools.jsp) [22]. *P* value < 0.05 was considered statistically significant.

### 2.4. Protein-Protein Interaction (PPI) Network and Module Analysis

An online tool, the Search Tool for the Retrieval of Interacting Genes (STRING) database (http://string.embl.de/) [23] was used to access the association of DEGs and integrate the PPI network. Interaction score > 0.4 was selected as statistically significant. Subsequently, the PPI network was visualized by the Cytoscape software [24]. The cytoHubba plugin and clusterONE plugin in Cytoscape were performed to identify hub genes and screen modules of the PPI network with the defaults [25, 26]. GO enrichment terms of hub genes and genes in modules were also analyzed by Metascape.

### 2.5. Statistical Analysis

All the statistical analyses were conducted with SPSS version 20.0 and GraphPad Prism version 8.0. The *t*-test was used to evaluate the gene expression level between DNMT3A mutation and wild-type AML. Chi-square analysis was used to evaluate the relationship between DNMT3 mutation and clinicopathological parameters. The Kaplan-Meier method with the log-rank test was used to calculate the overall survival (OS) of patients. Hazard ratio (HR) and 95% confidence intervals (CIs) were analyzed by the Cox proportional hazards regression model. *P* value < 0.05 was indicated as statistically significant.

## 3. Results

### 3.1. Data Source

A total of 170 AML patients were enrolled in the article from TCGA database. The inclusion criteria were as follows: (1) age ≥ 18 years old, (2) providing information of DNMT3A mutation, and (3) covering RNA-seq dataset and corresponding survival profiles and clinicopathological parameters. There were 43 AML patients with DNMT3A mutation (25%).

### 3.2. Survival Analysis

**Figure 1:**
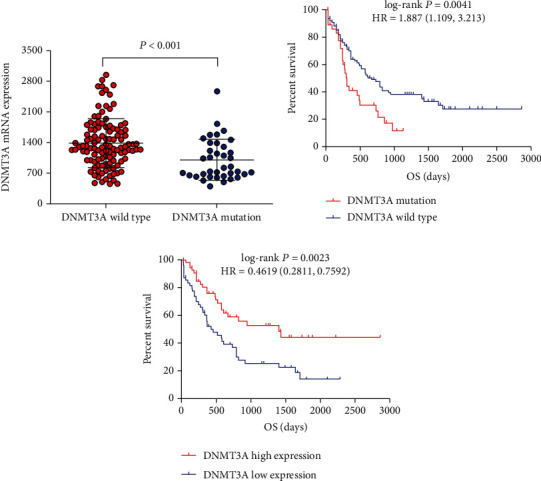
The comparison of mRNA expression and survival between DNMT3A mutation and wild-type AML. (a) The mRNA expression of the DNMT3A gene in AML patients' bone marrow tissue between DNMT3A with mutations and wild type. (b) Kaplan-Meier survival curves for AML patients stratified by DNMT3A mutation. (c) Kaplan-Meier survival curves for DNMT3A wild-type AML patients with different DNMT3A expression levels. AML: acute myeloid leukemia; OS: overall survival.

We observed that DNMT3A mRNA expression of bone marrow was different between DNMT3A mutation and wild-type AML patients (*P* < 0.001, Figure 1(a)). Survival analysis showed that patients with DNMT3A had a poor overall survival (log-rank *P* = 0.004). And univariate Cox proportional hazards regression analysis observed that DNMT3A mutation was correlated with poor prognosis and an increased risk of death (log-rank *P* = 0.004, HR = 2.216, 95%CI = 1.287, 3.816, Figure 1(b)). Excluding the mutant patients, we defined DNMT3A wild-type patients into the DNMT3A high expression (50%) and low expression (50%). The result showed that DNMT3A high expression was associated with favorable prognosis in AML patients (log-rank *P* = 0.002, HR = 0.462, 95%CI = 0.281, 0.759, Figure 1(c)).

### 3.3. Association between DNMT3 Mutation and Clinicopathological Parameters

To further understand the role of DNMT3 mutation in AML, chi-square analysis was used to evaluate the relationship between DNMT3 mutation and clinicopathological parameters (Table 1). The result indicated that DNMT3A mutation was obviously correlated with age (*P* < 0.001), blast percentage in peripheral blood (*P* = 0.006), and FLT3 mutation (*P* = 0.034). However, there was no statistical difference between DNMT3A mutation and sex (*P* = 0.862), FAB type (*P* = 0.326), blast percentage in bone marrow (*P* = 0.28), and white blood cell (*P* = 0.475).

### 3.4. Identification of DEGs

**Figure 2:**
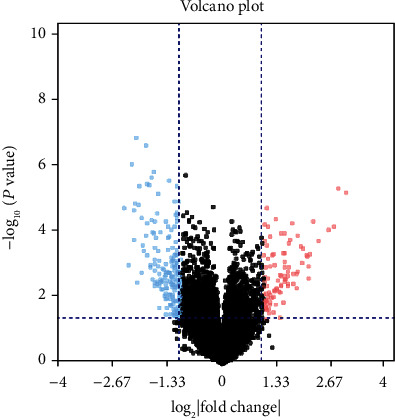
Volcano plot for differentially expressed genes. Black: nondifferentially expressed genes; red: upregulated differentially expressed genes; blue: downregulated differentially expressed genes.

Expected as a negative prognostic factor, it was important to understand how DNMT3A mutation influences the progress and function in AML. We screened DEGs of RNA-seq between 43 DNMT3A mutation and 127 wild-type AML patients with the criteria of log_2_  | fold change (FC) | ≥1 and *P* value < 0.05. Altogether, 283 DEGs were detected, which 95 were upregulated and 188 were downregulated (Table S1). The volcano plot of the DEGs is shown in Figure 2, and the heat map is shown in Figure S1.

### 3.5. GO and KEGG Enrichment Analyses

**Figure 3:**
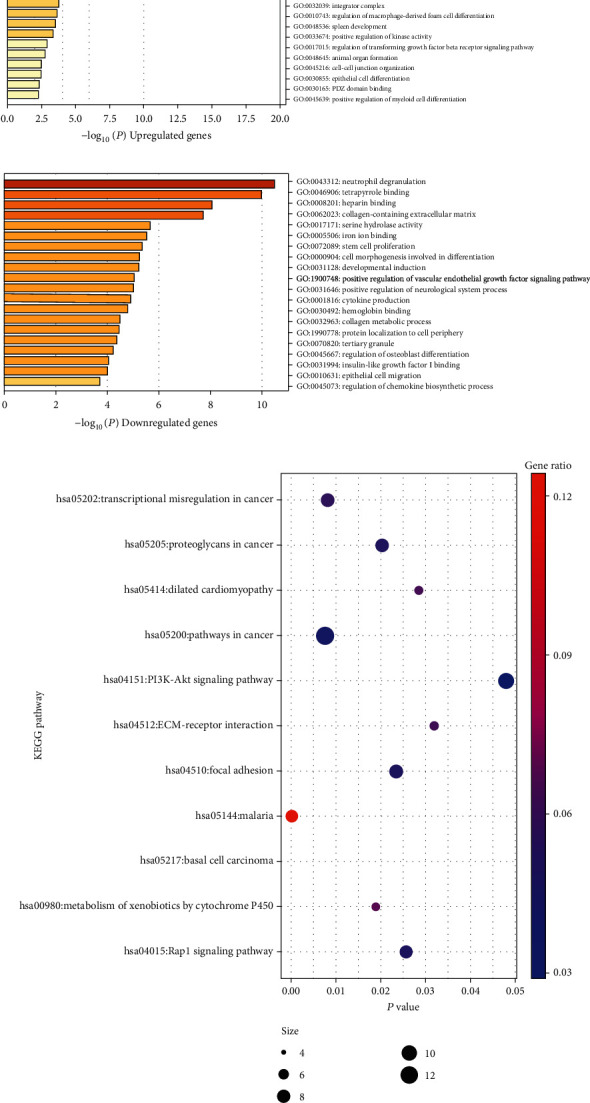
The top 20 GO enrichment terms and KEGG enrichment pathways of differentially expressed genes. (a) The top 20 GO enrichment terms of upregulated differentially expressed genes. (b) The top 20 GO enrichment terms of downregulated differentially expressed genes. (c) The KEGG enrichment pathways of differentially expressed genes. GO: Gene Ontology; KEGG: Kyoto Encyclopedia of Genes and Genomes; ECM: extracellular matrix.

283 DEGs were submitted for GO and KEGG pathway analyses with Metascape and DAVID, respectively. For biological processes, upregulated DEGs suggested significant enrichment in neutrophil degranulation, cell morphogenesis involved in differentiation, cytokine production, protein localization to cell periphery, epithelial cell migration, stem cell proliferation, collagen metabolic process, regulation of osteoblast differentiation, positive regulation of neurological system process, developmental induction, positive regulation of vascular endothelial growth factor signaling pathway, and regulation of chemokine biosynthetic process. For cellular components and molecular function, DEGs were enriched in collagen-SAZ/hydrolase activity, iron ion binding, hemoglobin binding, and insulin-like growth factor I binding (Figure 3(a), Table S2).

However, biological process enrichment showed downregulated DEGs were significantly enriched in embryonic skeletal system development, myeloid cell differentiation, leukocyte migration, tissue morphogenesis, gland development, positive regulation of kinase activity, epithelial cell differentiation, G protein-coupled receptor signaling pathway, coupled to cyclic nucleotide second messenger, definitive hemopoiesis, cell-cell junction organization, rhombomere development, uterus development, regulation of macrophage-derived foam cell differentiation, spleen development, animal organ formation, and positive regulation of myeloid cell differentiation. Cellular component and molecular function enrichment suggested DEGs were enriched in platelet alpha granule, integrator complex, and PDZ domain binding (Figure 3(b), Table S3).

KEGG pathway analysis was also conducted for total DEGs. The result showed DEGs were obviously enriched in pathways in cancer, PI3K-Akt signaling pathway, transcriptional misregulation in cancer, proteoglycans in cancer, focal adhesion, and Rap1 signaling pathway (Figure 3(c), Table S4).

### 3.6. PPI Network and Module Analysis

**Figure 4:**
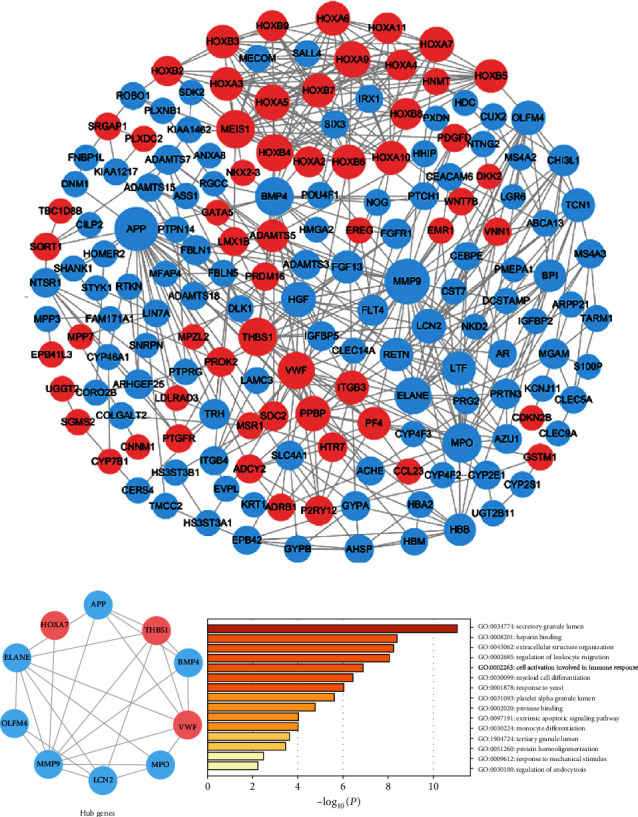
The protein-protein interactome (PPI) network and hub genes. (a) PPI network of differentially expressed genes. Red nodes mean upregulated differentially expressed genes, and blue nodes mean downregulated differentially expressed genes. The size of nodes means a combined degree of genes. (b) The PPI network and the GO term analysis of the top 10 hub genes. GO: Gene Ontology.

**Figure 5:**
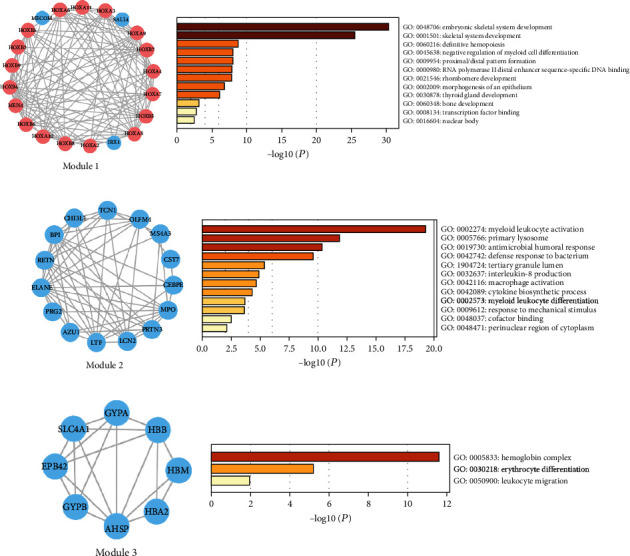
Module analysis of differentially expressed genes. Seven modules screened from the PPI network by the clusterONE plugin of Cytoscape. The genes of the top 3 modules ranked by *P* value were performed using GO enrichment analysis. Red nodes mean upregulated differentially expressed genes, and blue nodes mean downregulated differentially expressed genes. PPI: protein-protein interaction; GO: Gene Ontology.

To evaluate the association and hub genes of DEGs, the protein-protein interactome network was performed using the STRING and Cytoscape software (Figure 4(a)). We use 12 algorithms in cytoHubba plugin to detect the top 20 hub genes (Table S5). Furthermore, detected by more than 6 algorithms, the 10 hub genes interacted closer were selected to build the hub gene PPI network (Figure 4(b)). These 10 hub genes included ELANE, APP, MMP9, BMP4, MPO, THBS1, VWF, OLFM4, LCN2, and HOXA7. GO analysis by Metascape suggested hub genes significantly enriched in the regulation of leukocyte migration, myeloid cell differentiation, extrinsic apoptotic signaling pathway, and monocyte differentiation, which was associated with cancer (Figure 4(b)). Seven modules in the PPI network were detected by the clusterONE plugin (*P* < 0.05). We selected the top 3 modules to further analyze (Figure 5). The enrichment analysis demonstrated that the genes of module 1 were mainly enriched in embryonic skeletal system development, definitive hemopoiesis, and negative regulation of myeloid cell differentiation. The genes of module 2 were significantly enriched in myeloid leukocyte activation. In addition, GO analysis of module 3 genes was related to hemoglobin complex, erythrocyte differentiation, and leukocyte migration.

### 3.7. Expression Level and Prognostic Value of 10 Hub Genes

**Figure 6:**
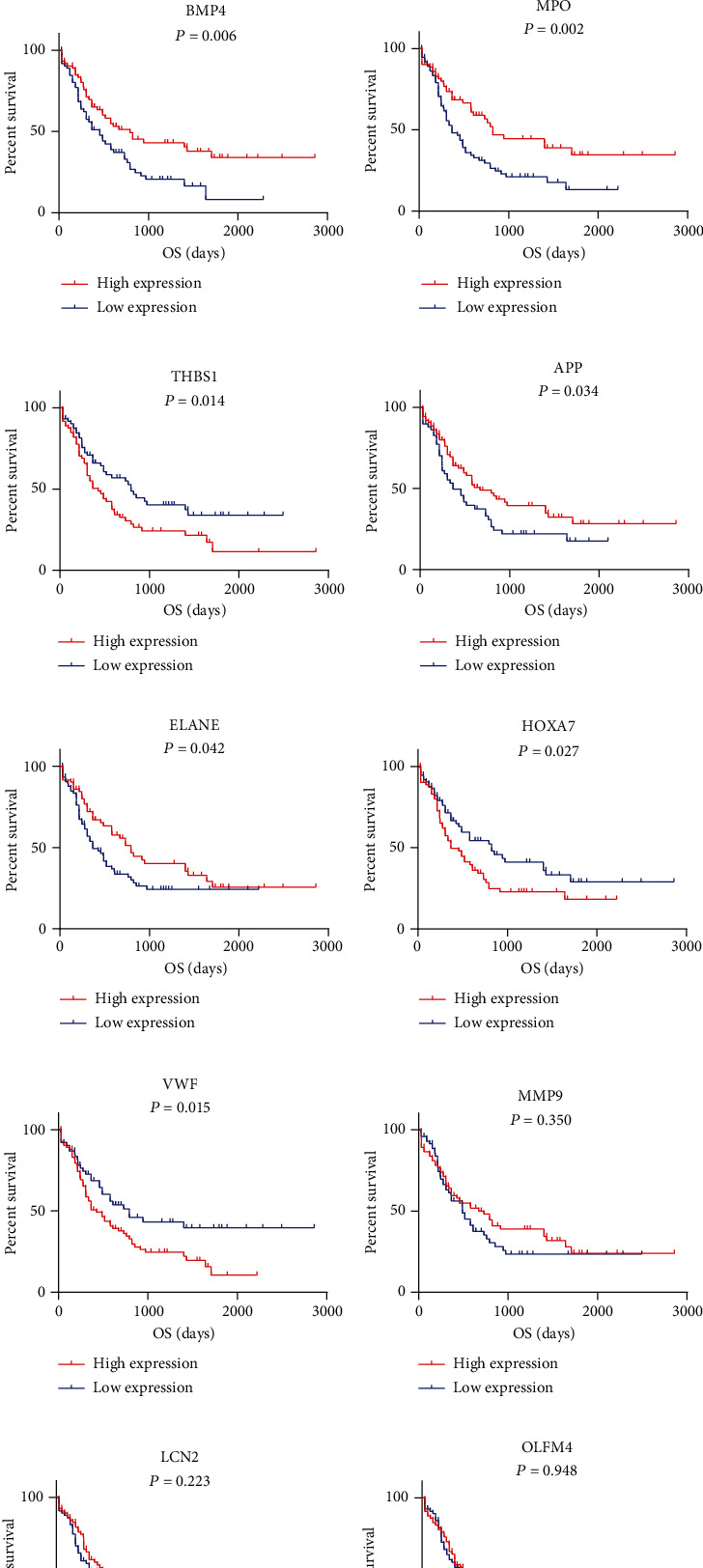
Expression level and prognostic value of 10 hub genes. (a) The expression level of 10 hub genes (ELANE, APP, MMP9, BMP4, MPO, THBS1, VWF, OLFM4, LCN2, and HOXA7) in DNMT3A mutation and wild-type patients. (b) Survival analyses for patients with different expression levels of above 10 hub genes. OS: overall survival.

The expression level of 10 hub genes (ELANE, APP, MMP9, BMP4, MPO, THBS1, VWF, OLFM4, LCN2, and HOXA7) in DNMT3A mutation and wild-type AML patients is shown in Figure 6(a). Corresponding survival analyses suggested that BMP4 (*P* = 0.006), MPO (*P* = 0.002), THBS1 (*P* = 0.014), APP (*P* = 0.034), ELANE (*P* = 0.042), HOXA7 (*P* = 0.027), and VWF (*P* = 0.015) had a significant prognostic value in AML (Figure 6).

## 4. Discussion

Multiple genetic alterations with a prognostic value can be suggested as biomarkers for AML to improve diagnosis and treatments. DNMT3A high-frequency mutation has been reported as a dangerous element in AML. DMNT3A might be a novel prognostic factor and therapeutic target of AML. Our study showed that DNMT3A expression was lower in wild-type AML compared with DNMT3A mutation AML. Survival analysis indicated that mutated leukemia patients had a shorter overall survival (OS) and increased risk of poor clinical outcome. This result was consistent with previous studies [17, 18, 27]. Dai et al. used the DNMT3A R878H conditional knock-in mouse model to predict specific lncRNAs regulated by the DNMT3A mutation in AML [28]. Yang et al. indicated that there were different clinical features and disease prognoses in AML patients with different DNMT3A mutation types, which were related to unique miRNA expression patterns. Moreover, the expression level of three miRNAs (miR-10b, miR-143, and miR-30b) was decreased in the DNMT3A R882 group [29]. In the present study, DNMT3A mutation was obviously associated with age, blast in peripheral blood, and FLT3 mutation.

However, the changes in biological processes and signal pathway DNMT3A mutation cause had not been reported. Herein, we used an RNA-seq dataset of adult AML from TCGA database to identify the key genes and pathways associated with DNMT3A mutation via bioinformatics analysis. Altogether, 283 DEGs were detected, which 95 were upregulated and 188 were downregulated. GO analyses showed that upregulated and downregulated DEGs were notably abundant in neutrophil degranulation, cell morphogenesis involved in differentiation, stem cell proliferation, myeloid cell differentiation, leukocyte migration, tissue morphogenesis, definitive hemopoiesis, and positive regulation of myeloid cell differentiation. It was suggested that DNMT3A mutations may contribute to disease progression and affect prognosis by influencing cell proliferation, differentiation, morphogenesis, and hemopoiesis in AML patients. The KEGG enrichment analysis revealed that DEGs were enriched in pathways of cancer, PI3K-Akt signaling pathway, and transcriptional misregulation in cancer. Consistent with previous studies, the above pathways have been reported that affect the pathogenesis and prognosis of AML [30–33].

What is more, we built the protein-protein interactome networks and selected some hub genes with high connectivity involved in DNMT3A mutation AML. The results about GO enrichment analysis of modules were similar to previous analysis of DEGs. The genes of the top 3 modules were enriched in hemopoiesis, myeloid cell differentiation, myeloid leukocyte activation, and migration which were closely associated with the pathogenesis of AML. The top hub genes included ELANE, APP, MMP9, BMP4, MPO, THBS1, VWF, OLFM4, LCN2, and HOXA7, which significantly enriched in regulation of leukocyte migration, myeloid cell differentiation, extrinsic apoptotic signaling pathway, and monocyte differentiation, associated with cancer. Corresponding survival analyses suggested that seven genes (BMP4, MPO, THBS1, APP, ELANE, HOXA7, and VWF) had a significant prognostic value in AML. Vandenberghe and Beel reported that ELANE mutation associated with severe congenital neutropenia increased the risk of AML [34]. And ELANE has been identified as a novel methylation prognostic signatures for clear cell renal cell carcinoma [35]. Researches have revealed that APP as a novel clue was involved in leukemia cell proliferation, extramedullary infiltration, and prognosis in AML [36, 37]. Azevedo et al. observed that changes in BMP4 expression regulated by the WNT canonical signaling pathway may be a potential mechanism of leukemogenesis [38]. Binato et al. also found that the decreasing expression of BMP4 in AML patients was related to the leukemogenic process [39]. The percentage of MPO-positive blast cells was regarded as a simple and highly significant prognostic factor in AML patients [40]. Tominaga-Sato et al. suggested higher MPO expression was associated with better overall survival after intensive chemotherapy [41]. THBS1 induces apoptosis of leukemia cells and could be a potential therapeutic target for AML patients [42, 43]. VWF participated in the processes of blood clotting and bleeding [44]. An interstitial deletion of a DNA segment between VWF and KRAS2 on der(12) was identified in AML-M1 [45]. In MLL-AF9-related leukemia, HOXA7 gene expression was potentially involved in the differentiation blockage [46]. These genes, mostly involved in the leukemia process, were potentially regarded as novel therapeutic targets in AML associated with DNMT3A mutation. Considering the small size of samples from TCGA database, further researches are needed to confirm our results.

## 5. Conclusion

Our study indicated that mutated DNMT3A resulted in a shorter OS which was in line with previous reports. Bioinformatics analyses showed DNMT3A mutation may contribute to disease progression and affect prognosis by influencing cell proliferation, differentiation, morphogenesis, and hemopoiesis in AML patients. Cancer pathway, PI3K-Akt signaling pathway, and transcriptional misregulation in cancer may play a crucial role in DNMT3A mutation AML. Seven hub genes (BMP4, MPO, THBS1, APP, ELANE, HOXA7, and VWF) had a significant prognostic value in AML. These findings provided novel targets for early diagnosis and new strategies for treatment for AML associated with DNMT3A mutation. But further experiments are still needed to support our results.

## Figures and Tables

**Table 1:** Association between DNMT3 mutation and clinicopathological parameters.

Clinicopathological variables	Cases (170)	DNMT3A		*P* value
		Mutation (43)	Wild type (127)	
Sex				
Male	89	22	67	0.862
Female	81	21	60	
Age (years)				
<60	89	43	46	<0.001
≥60	81	0	81	
FAB type				
M0	15	3	12	0.326
M1	44	12	32	
M2	38	12	26	
M3	16	7	9	
M4	32	4	28	
M5	18	4	14	
M6	2	0	2	
M7	3	0	3	
Unknown	2	1	1	
Blast in BM				
<50%	34	6	28	0.28
≥50%	136	37	99	
Blast in PB				
<50%	99	17	82	0.006
≥50%	68	25	43	
Unknown	3	1	2	
WBC (×10^9^/L)				
<10	68	15	53	0.475
≥10	102	28	74	
FLT3 mutation				
Yes	49	18	31	0.034
No	121	25	96	

Blast in BM: blast percentage in bone marrow; blast in PB: blast percentage in peripheral blood; WBC: white blood cell.

## Data Availability

The data used to support the findings of this study are available from the corresponding authors upon request.

## Supplementary Materials

Supplementary Materials Table S1: identification of differentially expressed genes (DEGs) between DNMT3A mutation and wild-type AML. Table S2: GO analysis of upregulated DEGs in AML with DNMT3A mutation. Table S3: GO analysis of downregulated DEGs in AML with DNMT3A mutation. Table S4: KEGG pathway analysis of DEGs in AML with DNMT3A mutation. Table S5: 20 hub genes analyzed by 12 different algorithms in Cytoscape. Figure S1: heat map of differentially expressed genes. Red: upregulation; green: downregulation.
