# Supplementary material for: Bioinformatics Analysis Identifies Key Genes and Pathways in Acute Myeloid Leukemia Associated with DNMT3A Mutation
Source: Biomed Res Int. 2020 Nov 23;2020:9321630. doi: 10.1155/2020/9321630 (PMC7707947; doi:10.1155/2020/9321630)
Supplement: Supplementary Materials — Table S1: identification of differentially expressed genes (DEGs) between DNMT3A mutation and wild-type AML. Table S2: GO analysis of upregulated DEGs in AML with DNMT3A mutation. Table S3: GO analysis of downregulated DEGs in AML with DNMT3A mutation. Table S4: KEGG pathway analysis of DEGs in AML with DNMT3A mutation. Table S5: 20 hub genes analyzed by 12 different algorithms in Cytoscape. Figure S1: heat map of differentially expressed genes. Red: upregulation; green: downregulation. [file 9321630.f1.zip › Table S1.docx]

| Differentially expressed genes (DEGs) | |
| --- | --- |
| Upregulated | Downregulated |
| HOXB6, HOXB5, PRDM16, NKX2-3, HOXA5, FLJ42875, HOXA6, HOXA9, HOXA7, HOXB3, HOXA3, HOXB4, C20orf200, HOXA4, WNT7B, HOXB9, MEIS1, CPNE8, CT45A1, HOXA10, TSPYL5, HOXB7, HOXB2, HOXA11AS, H2AFY2, UGGT2, EPB41L3, VNN1, L3MBTL4, C7orf58, SMPDL3A, LMX1B, HTR7, HOXA11, PPBP, CNNM1, ADRB1, EREG, LDLRAD3, ADCY2, VWF, CYP7B1, GSTM1, PROK2, HOXB8, SYTL4, HNMT, THBS1, TOM1L1, SAGE1, SRGAP1, CT45A3, SORT1, FAM190A, LOC728606, C17orf55, LOC100271722, PRICKLE2, C8orf79, PF4, PDGFD, PTGFR, P2RY12, MED12L, TMEM105, MSR1, HOXA2, MPZL2, SPATS2L, SDC2, GATA5, SLITRK4, C1QL1, CCL23, FAM81B, DTNA, CACNG4, SGMS2, CDKN2B, PPAPDC3, ONECUT2, TBC1D8B, DKK2, ITGB3, XIRP2, ADAMDEC1, PLXDC2, MARVELD2, TM7SF4, ADAMTS5, HNRPLL, LOC441204, GLB1L2, EMR1, MPP7  (n=95) | ZNF415, LAMC3, NKD2, BLID, OLFM4, CYP2E1, ADAMTS18, ADAMTS7, EBF3, NPM2, DOC2B, GSTT1, CLEC9A, PAR-SN, LGR6, IGDCC4, C1orf21, ROBO1, SNORD116-20, LCN2, SLC26A9, ADAMTS15, FNBP1L, C13orf15, LOC154822, IL5RA, FAM134B, HBM, NTNG2, PTPRG, HMGA2, SAMD11, SLC28A3, DNM1, RNF165, AZU1, GEFT, TCN1, ZSCAN23, FBLN1, C9orf122, CORO2B, EPB42, IGFBP5, SNRPN, RPS6KA2, STYK1, FBLN5, HS3ST3B1, C1orf228, FGF13, PLXNB1, PRTN3, PMEPA1, LIN7A, IFITM1, C2orf65, MPP3, ASS1, SHANK3, KCNJ11, MECOM, HS3ST3A1, FUT1, CECR6, CLDN9, IL12RB2, CASKIN2, AR, HBB, DAGLA, ACHE, LOC100130264, PPP1R14A, APP, BMP4, OR9A4, HDX, IPW, DUSP27, GYPB, HHIP, GLT25D2, CD200, AHSP, ADAMTS3, CST7, STOX2, MGAM, CUX2, EBF4, DCHS1, PTCH1, CCDC8, UNCX, KIF26A, FAM171A1, LEPREL2, RAB3IL1, TMCC2, PRG2, SLC29A4, KIAA1462, GYPA, FLT4, CYP4F3, IGFBP2, ARPP21, SLC4A1, EVPL, RTKN, ZNF608, ABCA13, CLIP3, TARM1, CILP2, ITGB4, SNORD116-4, SDK2, GPR12, TSPAN7, UPK3BL, PCBP3, C1QTNF4, PTPN14, KRT1, KIAA1217, MEG3, CEBPE, S100P, HBA2, CLEC14A, HDC, CYP46A1, VPREB1, CEACAM6, PTPRM, CHI3L1, MPO, CDH4, MFAP4, SIX3, LTF, KIRREL, CYP4F2, UGT2B11, LRRC2, LPO, CACNA1H, POU4F1, SLCO5A1, NTSR1, ANXA8L2, TMIGD2, DDIT4L, ELANE, MMP9, BPI, CYP2S1, ATP9A, MGC16121, GABRD, SALL4, DLK1, LASS4, HGF, RETN, IRX1, AFF2, ANXA8, NOG, MS4A2, MS4A3, FGFR1, VSTM1, LOC399959, GABRE, CLEC5A, TPSB2, TPSAB1, PXDN, PALM, LOXHD1, SHANK1, TPSD1, PRRT4, TRH, UGT3A2  (n=188) |

**Table S1 Identification of differentially expressed genes (DEGs) between DNMT3A mutation and wild-type AML**
