# Supplementary material for: Bioinformatics Analysis Identifies Key Genes and Pathways in Acute Myeloid Leukemia Associated with DNMT3A Mutation
Source: Biomed Res Int. 2020 Nov 23;2020:9321630. doi: 10.1155/2020/9321630 (PMC7707947; doi:10.1155/2020/9321630)
Supplement: Supplementary Materials — Table S1: identification of differentially expressed genes (DEGs) between DNMT3A mutation and wild-type AML. Table S2: GO analysis of upregulated DEGs in AML with DNMT3A mutation. Table S3: GO analysis of downregulated DEGs in AML with DNMT3A mutation. Table S4: KEGG pathway analysis of DEGs in AML with DNMT3A mutation. Table S5: 20 hub genes analyzed by 12 different algorithms in Cytoscape. Figure S1: heat map of differentially expressed genes. Red: upregulation; green: downregulation. [file 9321630.f1.zip › Table S2.docx]

| **GO** | **Category** | **Description** | **Count** | **%** | **Log10(P)** | **Log10(q)** |
| --- | --- | --- | --- | --- | --- | --- |
| GO:0048706 | GO Biological Processes | embryonic skeletal system development | 16 | 17.02 | -19.44 | -15.09 |
| GO:0030099 | GO Biological Processes | myeloid cell differentiation | 11 | 11.7 | -6.16 | -3.06 |
| GO:0050900 | GO Biological Processes | leukocyte migration | 11 | 11.7 | -5.4 | -2.46 |
| GO:0048729 | GO Biological Processes | tissue morphogenesis | 11 | 11.7 | -4.21 | -1.41 |
| GO:0048732 | GO Biological Processes | gland development | 9 | 9.57 | -4.3 | -1.48 |
| GO:0033674 | GO Biological Processes | positive regulation of kinase activity | 9 | 9.57 | -3.39 | -0.8 |
| GO:0030855 | GO Biological Processes | epithelial cell differentiation | 9 | 9.57 | -2.47 | -0.1 |
| GO:0007187 | GO Biological Processes | G protein-coupled receptor signaling pathway, coupled to cyclic nucleotide second messenger | 7 | 7.45 | -4.18 | -1.4 |
| GO:0031091 | GO Cellular Components | platelet alpha granule | 6 | 6.38 | -5.82 | -2.83 |
| GO:0060216 | GO Biological Processes | definitive hemopoiesis | 4 | 4.26 | -6 | -2.95 |
| GO:0017015 | GO Biological Processes | regulation of transforming growth factor beta receptor signaling pathway | 4 | 4.26 | -2.89 | -0.38 |
| GO:0045216 | GO Biological Processes | cell-cell junction organization | 4 | 4.26 | -2.48 | -0.1 |
| GO:0021546 | GO Biological Processes | rhombomere development | 3 | 3.19 | -5.7 | -2.73 |
| GO:0060065 | GO Biological Processes | uterus development | 3 | 3.19 | -4.08 | -1.33 |
| GO:0032039 | GO Cellular Components | integrator complex | 3 | 3.19 | -3.76 | -1.05 |
| GO:0010743 | GO Biological Processes | regulation of macrophage derived foam cell differentiation | 3 | 3.19 | -3.67 | -1.02 |
| GO:0048536 | GO Biological Processes | spleen development | 3 | 3.19 | -3.54 | -0.93 |
| GO:0048645 | GO Biological Processes | animal organ formation | 3 | 3.19 | -2.76 | -0.31 |
| GO:0030165 | GO Molecular Functions | PDZ domain binding | 3 | 3.19 | -2.32 | 0 |
| GO:0045639 | GO Biological Processes | positive regulation of myeloid cell differentiation | 3 | 3.19 | -2.25 | 0 |

**Table S2 GO analysis of upregulated DEGs in AML with DNMT3A mutation**

GO, gene ontology, DEGs, differentially expressed genes, AML, acute myeloid leukemia.
