# Supplementary material for: Bioinformatics Analysis Identifies Key Genes and Pathways in Acute Myeloid Leukemia Associated with DNMT3A Mutation
Source: Biomed Res Int. 2020 Nov 23;2020:9321630. doi: 10.1155/2020/9321630 (PMC7707947; doi:10.1155/2020/9321630)
Supplement: Supplementary Materials — Table S1: identification of differentially expressed genes (DEGs) between DNMT3A mutation and wild-type AML. Table S2: GO analysis of upregulated DEGs in AML with DNMT3A mutation. Table S3: GO analysis of downregulated DEGs in AML with DNMT3A mutation. Table S4: KEGG pathway analysis of DEGs in AML with DNMT3A mutation. Table S5: 20 hub genes analyzed by 12 different algorithms in Cytoscape. Figure S1: heat map of differentially expressed genes. Red: upregulation; green: downregulation. [file 9321630.f1.zip › Table S3.docx]

| **GO** | **Category** | **Description** | **Count** | **%** | **Log10(P)** | **Log10(q)** |
| --- | --- | --- | --- | --- | --- | --- |
| GO:0043312 | GO Biological Processes | neutrophil degranulation | 22 | 11.83 | -10.53 | -6.65 |
| GO:0000904 | GO Biological Processes | cell morphogenesis involved in differentiation | 19 | 10.22 | -5.28 | -2.58 |
| GO:0001816 | GO Biological Processes | cytokine production | 19 | 10.22 | -4.9 | -2.27 |
| GO:0062023 | GO Cellular Components | collagen-containing extracellular matrix | 17 | 9.14 | -7.72 | -4.64 |
| GO:0046906 | GO Molecular Functions | tetrapyrrole binding | 13 | 6.99 | -9.98 | -6.47 |
| GO:0008201 | GO Molecular Functions | heparin binding | 12 | 6.45 | -8.09 | -4.92 |
| GO:1990778 | GO Biological Processes | protein localization to cell periphery | 11 | 5.91 | -4.48 | -1.93 |
| GO:0010631 | GO Biological Processes | epithelial cell migration | 11 | 5.91 | -4.01 | -1.59 |
| GO:0017171 | GO Molecular Functions | serine hydrolase activity | 10 | 5.38 | -5.68 | -2.9 |
| GO:0005506 | GO Molecular Functions | iron ion binding | 9 | 4.84 | -5.54 | -2.78 |
| GO:0072089 | GO Biological Processes | stem cell proliferation | 8 | 4.3 | -5.37 | -2.64 |
| GO:0070820 | GO Cellular Components | tertiary granule | 8 | 4.3 | -4.38 | -1.84 |
| GO:0032963 | GO Biological Processes | collagen metabolic process | 7 | 3.76 | -4.51 | -1.94 |
| GO:0045667 | GO Biological Processes | regulation of osteoblast differentiation | 7 | 3.76 | -4.25 | -1.76 |
| GO:0031646 | GO Biological Processes | positive regulation of neurological system process | 6 | 3.23 | -5.04 | -2.38 |
| GO:0031128 | GO Biological Processes | developmental induction | 5 | 2.69 | -5.23 | -2.55 |
| GO:1900748 | GO Biological Processes | positive regulation of vascular endothelial growth factor signaling pathway | 3 | 1.61 | -5.05 | -2.38 |
| GO:0030492 | GO Molecular Functions | hemoglobin binding | 3 | 1.61 | -4.81 | -2.22 |
| GO:0031994 | GO Molecular Functions | insulin-like growth factor I binding | 3 | 1.61 | -4.03 | -1.6 |
| GO:0045073 | GO Biological Processes | regulation of chemokine biosynthetic process | 3 | 1.61 | -3.72 | -1.39 |

**Table S3 GO analysis of downregulated DEGs in AML with DNMT3A mutation**

GO, gene ontology, DEGs, differentially expressed genes, AML, acute myeloid leukemia.
