# Supplementary material for: Bioinformatics Analysis Identifies Key Genes and Pathways in Acute Myeloid Leukemia Associated with DNMT3A Mutation
Source: Biomed Res Int. 2020 Nov 23;2020:9321630. doi: 10.1155/2020/9321630 (PMC7707947; doi:10.1155/2020/9321630)
Supplement: Supplementary Materials — Table S1: identification of differentially expressed genes (DEGs) between DNMT3A mutation and wild-type AML. Table S2: GO analysis of upregulated DEGs in AML with DNMT3A mutation. Table S3: GO analysis of downregulated DEGs in AML with DNMT3A mutation. Table S4: KEGG pathway analysis of DEGs in AML with DNMT3A mutation. Table S5: 20 hub genes analyzed by 12 different algorithms in Cytoscape. Figure S1: heat map of differentially expressed genes. Red: upregulation; green: downregulation. [file 9321630.f1.zip › Table S4.docx]

| Pathway ID | Name | Count | % | P Value | Genes |
| --- | --- | --- | --- | --- | --- |
| hsa05200 | Pathways in cancer | 13 | 0.038934 | 0.007524 | BMP4, FGFR1, AR, ADCY2, MMP9, FGF13, HGF, MECOM, WNT7B, CDKN2B, LAMC3, PTCH1, HHIP |
| hsa04151 | PI3K-Akt signaling pathway | 10 | 0.029949 | 0.04795 | FGFR1, VWF, LAMC3, FLT4, ITGB4, FGF13, PDGFD, HGF, ITGB3, THBS1 |
| hsa05202 | Transcriptional misregulation in cancer | 8 | 0.023959 | 0.008082 | CEBPE, MMP9, ELANE, HOXA10, TSPAN7, MPO, HMGA2, MEIS1 |
| hsa05205 | Proteoglycans in cancer | 8 | 0.023959 | 0.020239 | FGFR1, WNT7B, MMP9, PTCH1, HGF, ITGB3, THBS1, SDC2 |
| hsa04510 | Focal adhesion | 8 | 0.023959 | 0.023369 | VWF, LAMC3, FLT4, ITGB4, PDGFD, HGF, ITGB3, THBS1 |
| hsa04015 | Rap1 signaling pathway | 8 | 0.023959 | 0.025633 | FGFR1, ADCY2, FLT4, FGF13, PDGFD, HGF, ITGB3, THBS1 |
| hsa05144 | Malaria | 7 | 0.020964 | 5.13E-05 | GYPB, GYPA, HBA2, HGF, THBS1, HBB, SDC2 |
| hsa00980 | Metabolism of xenobiotics by cytochrome P450 | 5 | 0.014975 | 0.018823 | GSTM1, CYP2S1, UGT2B11, GSTT1, CYP2E1 |
| hsa05414 | Dilated cardiomyopathy | 5 | 0.014975 | 0.028461 | ADCY2, ADRB1, ITGB4, CACNG4, ITGB3 |
| hsa04512 | ECM-receptor interaction | 5 | 0.014975 | 0.031833 | VWF, LAMC3, ITGB4, ITGB3, THBS1 |
| hsa05217 | Basal cell carcinoma | 4 | 0.01198 | 0.038142 | BMP4, WNT7B, PTCH1, HHIP |

**Table S4 KEGG pathway analysis of DEGs in AML with DNMT3A mutation**

KEGG, Kyoto Encyclopedia of Genes and Genomes, DEGs, differentially expressed genes, ECM, extracellular matrix.
