# Supplementary material for: Bioinformatics Analysis Identifies Key Genes and Pathways in Acute Myeloid Leukemia Associated with DNMT3A Mutation
Source: Biomed Res Int. 2020 Nov 23;2020:9321630. doi: 10.1155/2020/9321630 (PMC7707947; doi:10.1155/2020/9321630)
Supplement: Supplementary Materials — Table S1: identification of differentially expressed genes (DEGs) between DNMT3A mutation and wild-type AML. Table S2: GO analysis of upregulated DEGs in AML with DNMT3A mutation. Table S3: GO analysis of downregulated DEGs in AML with DNMT3A mutation. Table S4: KEGG pathway analysis of DEGs in AML with DNMT3A mutation. Table S5: 20 hub genes analyzed by 12 different algorithms in Cytoscape. Figure S1: heat map of differentially expressed genes. Red: upregulation; green: downregulation. [file 9321630.f1.zip › Table S5.docx]

| Rank | algorithms | | | | | | | | | | | |
| --- | --- | --- | --- | --- | --- | --- | --- | --- | --- | --- | --- | --- |
|  | Betweenness | BottleNeck | Closeness | Clustering Coefficient | Degree | DMNC | EcCentricity | EPC | MCC | MNC | Radiality | Stress |
| 1 | APP | APP | MMP9 | CCL23 | MMP9 | HOXB6 | AHSP | MMP9 | MEIS1 | MMP9 | MMP9 | APP |
| 2 | MMP9 | MMP9 | APP | DCSTAMP | APP | HOXB8 | HBA1 | VWF | HOXA5 | APP | APP | MMP9 |
| 3 | BMP4 | BMP4 | THBS1 | EMR1 | MPO | HOXB5 | HBB | APP | HOXA7 | THBS1 | VWF | BMP4 |
| 4 | MPO | ELANE | VWF | PRG2 | MEIS1 | HOXA4 | GYPA | MPO | HOXA6 | MPO | THBS1 | MPO |
| 5 | THBS1 | THBS1 | BMP4 | EREG | THBS1 | CHI3L1 | GYPB | THBS1 | HOXB4 | MEIS1 | BMP4 | THBS1 |
| 6 | ARHGEF25 | TCN1 | ELANE | PLXDC2 | BMP4 | HOXA6 | ALAS2 | ELANE | HOXB6 | VWF | PPBP | VWF |
| 7 | VWF | MPO | PPBP | CLEC14A | HOXB4 | HOXA7 | SLC4A1 | LTF | HOXB3 | ELANE | ELANE | ARHGEF25 |
| 8 | RTKN | ARHGEF25 | HGF | GYPB | VWF | HOXB3 | EPB42 | PPBP | HOXB5 | HOXB4 | HGF | ELANE |
| 9 | TRH | MGAM | MPO | PTGFR | ELANE | HOXA3 | HBG1 | HGF | HOXA9 | HOXA9 | LTF | ITGB3 |
| 10 | MGAM | VWF | LTF | ADAMTS15 | HOXA9 | HOXB2 | HOXB6 | LCN2 | HOXA3 | HOXA5 | PF4 | OLFM4 |
| 11 | LMX1B | HOXB6 | ITGB3 | ADAMTS18 | HOXA5 | HOXA5 | HBG2 | RETN | HOXB7 | PPBP | ITGB3 | TCN1 |
| 12 | LIN7A | TRH | PF4 | CYP4F2 | HGF | HOXA11 | HBA2 | BMP4 | HOXB8 | BMP4 | MPO | RTKN |
| 13 | ITGB3 | HOXB4 | FGF13 | CYP4F3 | HOXA7 | HOXA2 | APOB | OLFM4 | HOXA10 | LTF | FGF13 | HGF |
| 14 | TCN1 | RTKN | LCN2 | HS3ST3A1 | PPBP | HOXA10 | LCT | PF4 | HOXA4 | LCN2 | LCN2 | HOXA7 |
| 15 | ELANE | MEIS1 | OLFM4 | HS3ST3B1 | OLFM4 | PTGFR | RHCE | HOXA7 | HOXA11 | HOXA6 | PROK2 | LCN2 |
| 16 | OLFM4 | ITGB3 | TCN1 | MFAP4 | LTF | ADAMTS15 | HEMGN | MEIS1 | ELANE | HOXA7 | OLFM4 | TRH |
| 17 | HGF | HBB | HOXA7 | HBA2 | LCN2 | ADAMTS7 | MYL4 | HOXB4 | RETN | HOXA3 | TCN1 | FGF13 |
| 18 | CYP2E1 | AR | RETN | HOXA4 | HOXA6 | ARHGEF25 | HBM | HOXA5 | LTF | HOXB3 | RETN | PPBP |
| 19 | FBLN1 | ACHE | HOXB4 | HOXB8 | HOXB6 | ADAMTS18 | C7 | TCN1 | LCN2 | HOXB7 | ACHE | MGAM |
| 20 | ITGB4 | LIN7A | ADAMTS5 | HOXA11 | HOXA10 | TRH | SELENBP1 | ITGB3 | OLFM4 | RETN | IGFBP5 | LIN7A |

**Table S5 20 hub genes analyzed by 12 different algorithms in Cytoscape.**
