# Supplementary figures and images for: Bioinformatics Analysis Identifies Key Genes and Pathways in Acute Myeloid Leukemia Associated with DNMT3A Mutation
Source: Biomed Res Int. 2020 Nov 23;2020:9321630. doi: 10.1155/2020/9321630 (PMC7707947; doi:10.1155/2020/9321630)

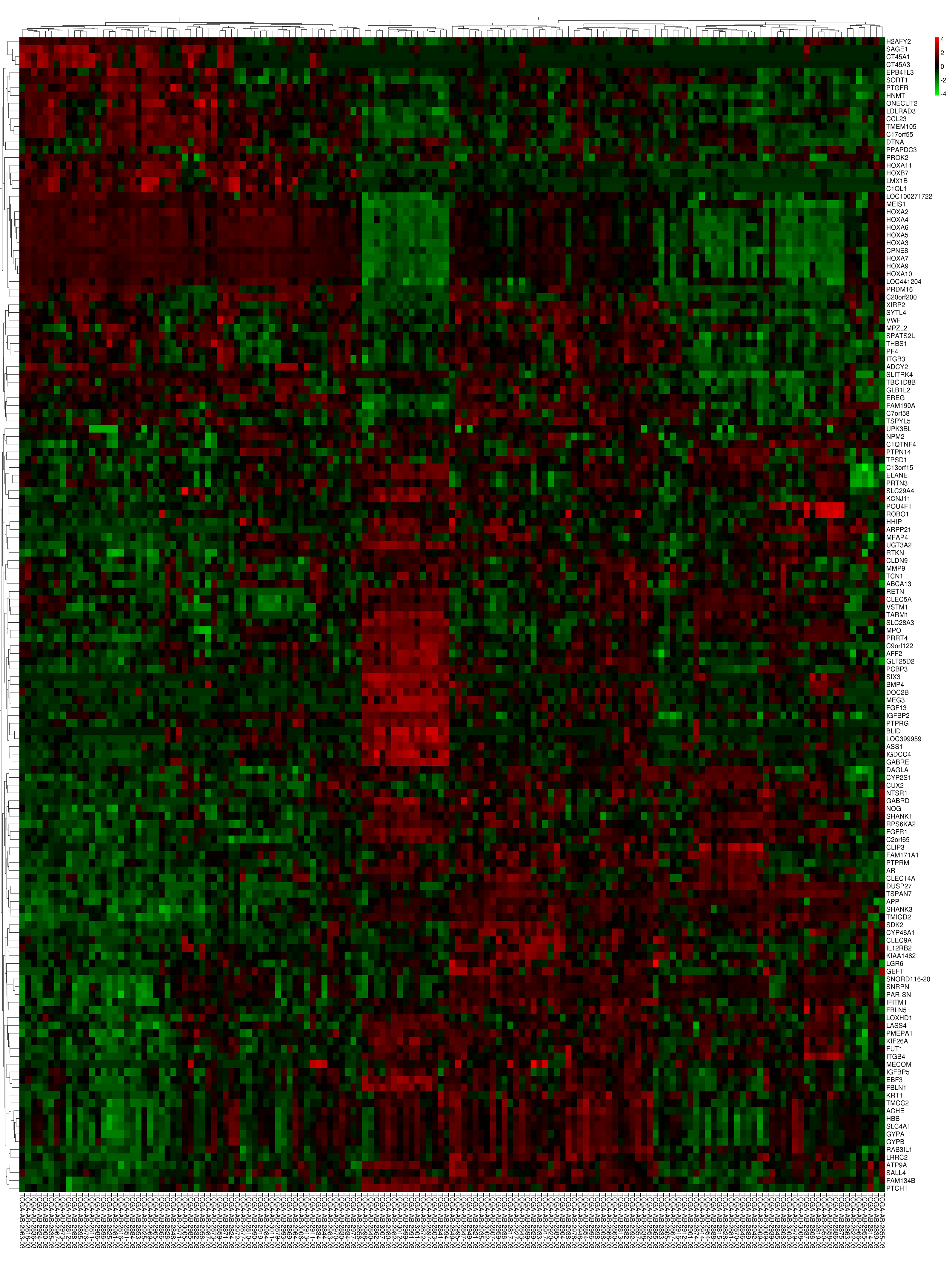

Supplement: Supplementary Materials — Table S1: identification of differentially expressed genes (DEGs) between DNMT3A mutation and wild-type AML. Table S2: GO analysis of upregulated DEGs in AML with DNMT3A mutation. Table S3: GO analysis of downregulated DEGs in AML with DNMT3A mutation. Table S4: KEGG pathway analysis of DEGs in AML with DNMT3A mutation. Table S5: 20 hub genes analyzed by 12 different algorithms in Cytoscape. Figure S1: heat map of differentially expressed genes. Red: upregulation; green: downregulation. [file 9321630.f1.zip › Fig S 1.tif]
